# Traumatic insemination and female counter-adaptation in Strepsiptera (Insecta)

**DOI:** 10.1038/srep25052

**Published:** 2016-04-29

**Authors:** Miriam Peinert, Benjamin Wipfler, Gottfried Jetschke, Thomas Kleinteich, Stanislav N. Gorb, Rolf G. Beutel, Hans Pohl

**Affiliations:** 1 Entomology Group, Institut für Spezielle Zoologie und Evolutionsbiologie mit Phyletischem Museum, Friedrich-Schiller-Universität Jena, Erbertstrasse 1, D-07743 Jena, Germany; 2 Institut für Ökologie, Friedrich-Schiller-Universität Jena, Dornburger Strasse 159, D-07743 Jena, Germany; 3 Department of Functional Morphology and Biomechanics, Institute of Zoology, Christian-Albrechts-Universität zu Kiel, Am Botanischen Garten 1–9, D-24118 Kiel, Germany

## Abstract

In a few insect groups, males pierce the female’s integument with their penis during copulation to transfer sperm. This so-called traumatic insemination was previously confirmed for Strepsiptera but only in species with free-living females. The more derived endoparasitic groups (Stylopidia) were suggested to exhibit brood canal mating. Further, it was assumed that females mate once and that pheromone production ceases immediately thereafter. Here we examined *Stylops ovinae* to provide details of the mating behaviour within Stylopidia. By using μCT imaging of *Stylops* in copula, we observed traumatic insemination and not, as previously suggested, brood canal mating. The penis is inserted in an invagination of the female cephalothorax and perforates its cuticle. Further we show that female *Stylops* are polyandrous and that males detect the mating status of the females. Compared to other strepsipterans the copulation is distinctly prolonged. This may reduce the competition between sperm of the first mating male with sperm from others. We describe a novel paragenital organ of *Stylops* females, the cephalothoracic invagination, which we suggest to reduce the cost of injuries. In contrast to previous interpretations we postulate that the original mode of traumatic insemination was maintained after the transition from free-living to endoparasitic strepsipteran females.

Copulation that involves the wounding of the mating partner by specialised devices is a widespread and diverse phenomenon in animals and evolved multiple times within different taxa. It occurs in nematodes, terrestrial arthropods, flatworms, rotifers, annelids, snails and slugs, and even in amphibians (for a recent review see Reinhardt *et al*.^1^). In arthropods, this bizarre mode of copulation, termed traumatic insemination, occurs in one spider (*Harpactea sadistica*, Dysderidae^2^) and fruit flies^3^,^4^, and it is prevalent and best investigated in cimicomorph bugs^5^. In this megadiverse lineage with approximately 25,000 species, traumatic insemination has evolved at least three times independently. It is assumed that traumatic insemination evolved due to sperm competition as a means for males bypassing the female genital tract and sperm storage organs, and thereby circumventing pre- and postcopulatory female choice mechanisms^5^. Only in Cimicomorpha, traumatic insemination is known to impose costs on the female. To mitigate these costs females have repeatedly evolved defensive paragenitalia, indicative of sexual conflict^6^. The twisted winged parasites (Strepsiptera) (ca. 600 described extant species) are the second largest group of terrestrial arthropods, which are reported to inseminate traumatically^5^. However, it was postulated recently that this is not the general mode of copulation in strepsipterans, but only occurs in the most basal group with free-living females. A secondary change to non-traumatic insemination in the majority of strepsipterans with permanent endoparasitic females was suggested^7^.

Strepsiptera are one of the most specialised insect orders. All species display extreme sexual dimorphism. The males are free living and the only purpose of their very short adult life of a few hours is to find a female to copulate. The females, with the exception of the basal Mengenillidae, and all larvae are obligatory parasites of other insects. In contrast to the males they are wingless and structurally strongly simplified, with an extremely reduced genital apparatus. The ovipositor, vagina, receptaculum seminis, genital chamber, bursa copulatrix, accessory glands and ovarioles are missing. The eggs float freely in the hemolymph. A single brood organ is present. It is not used for copulation but only for releasing the first instar larvae^8^. In all remaining strepsipterans (Stylopidia), the females are permanent endoparasites of winged insects, such as grasshoppers, true bugs or wasps, where they remain within the host’s abdomen with the larger posterior part of their body and only the anterior cephalothorax is exposed. Females, ventrally placed, are covered by overlapping larval exuviae. They are lacking antennae, eyes, most mouthparts, wings, legs and genitalia. The floor of the brood canal is lined by a cuticle and by the larval exuviae. It enables the first instar larvae to leave the female through the birth opening, which is also used for insemination^9^,^10^.

The unusual morphology and behaviour of strepsipterans led to different controversies in the literature. One of them was the systematic placement, which was termed “the Strepsiptera problem”^11^,^12^. Only recently Coleoptera were unambiguously confirmed as the sister group using morphological and phylogenomic approaches^13^,^14^,^15^,^16^. Another major unresolved question concerns the reproductive behaviour and copulatory mode, which are documented only fragmentarily and with conflicting results and interpretations. Originally, parthenogenesis was discussed based on the complete loss of the female genital apparatus and the alleged extraordinary rarity of males^17^,^18^. However Schrader^9^, provided the first definite proof for the presence of sexual reproduction. Early treatments of strepsipteran mating are only short notes on incidental observations^8^,^18^,^19^,^20^,^21^,^22^,^23^,^24^. These studies reported traumatic insemination of the free living females of the basal Mengenillidae^8^. In Stylopidia, however, the insertion of the penis in the female cephalothorax was confirmed, without clarifying the actual process of insemination. Some authors suggested traumatic insemination (e.g.^18^), whereas others postulated that the penis is only inserted in the brood canal, with the spermatozoa reaching the body cavity of the female by moving along the brood canal and birth organs (ectodermal invaginations connecting the brood canal with the hemocoel) (e.g.^9^).

Detailed studies on the copulation of Stylopidia were presented by Lauterbach^25^ and recently by Beani *et al.*^10^ and Hrabar *et al.*^26^. Lauterbach did not find spermatozoa in the brood canal and birth organs on histological sections of recently mated females. He observed darkly pigmented chitinous structures in the brood canal of females of *Stylops ovinae*, which were examined one day after copulation or later. He interpreted them as mating signs and therefore concluded traumatic insemination. Beani *et al.*^10^, who used scanning and transmission electron microscopy, identified spermatozoa not only in the hemocoel, but also in the brood canal and birth organs of recently mated females of *Xenos vesparum. Hrabar* *et al.*^26^, who focussed on the precopulatory and postcopulatory behaviour of females of *Xenos peckii*, provided a detailed description and high-speed video sequences of the copulation. However, the specific mode of copulation was not investigated. According to Beani *et al.*^10^, traumatic insemination in Stylopidia is an open question, as the spermatozoa can reach the oocytes either through the hemocoel as a result of this drastic mode of transfer, or alternatively by moving along the brood canal and birth organs. In a recent review^7^ it was suggested that traumatic insemination is replaced by brood canal mating in Stylopidia in correlation with endoparasitism. It was further assumed that only virgin females are attractive and that pheromone production ceases immediately after copulation and that mated females are no longer attractive to males^27^,^28^.

Our main aim was to document the mating of *S. ovinae* in detail using a combination of different modern techniques to deepen our knowledge on the evolutionary mechanisms behind the diversity of mating modes among insects. A major issue is the specific mode of sperm transfer, either as traumatic insemination, or alternatively an insertion of the penis into the brood canal without penetration of the body wall. Another focus is on the role of a paragenital structure and the assumed monandry of females of *S. ovinae*. In contrast to previous studies, we used an integrative approach. In addition to video recordings of the mating, the duration and frequency of copulation were assessed. Histological sections of virgin females and females fixed shortly after copulation were made. Micro-CT scans of fixed copulae of *S. ovinae* were taken and the involved structures were studied with scanning electron microscopy and reconstructed three-dimensionally.

## Results

### Female structures of *Stylops ovinae* associated with copulation

**Figure 1:**
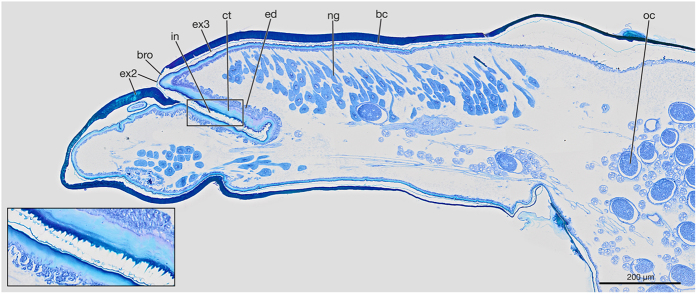
Slightly semilateral sagittal section of the cephalothorax and the anterior part of the abdomen of a virgin female of *S. ovinae*, anterior is toward the left and ventral (physiological dorsal) is toward the top. Inset: Detail of the cephalothoracic invagination showing the thickened cuticle and microtrichia. bc, brood canal; bro, birth opening; ct, cuticle; ed, epidermis; ex2, exuvium of second instar larva; ex3, exuvium of third instar larva; in, cephalothoracic invagination; ng, Nassonov glands; oc, egg.

The crescent-shaped birth opening is located posterior to the vestigial mouth parts. It leads into the lumen of the brood canal that extends ventrally through the cephalothorax and reaches abdominal segment VII posteriorly. The lumen of the brood canal is filled with air in living females. It is very narrow in the cephalothorax but widens in the abdomen (Fig. 1). A single birth organ is present in each of segments II–VII. The pheromone glands (Nassonov glands)^29^ are located in the cephalothorax and fill out most of its lumen (Fig. 1). They open into the lumen of the brood canal.

A transverse fissure-shaped and curved invagination is present directly in front of the birth opening. In virgin females its opening is covered by a thin cuticle along with the opening of the brood canal (Fig. 1). The invagination extends into the cephalothorax at an angle of about 60°. It is 280 μm long, approximately one-third of the length of the cephalothorax. The ventral cuticular surface of the invagination is densely covered with microtrichia (Fig. 1, inset). On the ventral side the cuticle is about three times as thick (47 μm) as on the dorsal side (15 μm). Additionally, the epidermis of the ventral side is multi-layered.

### External male genital structures of *Stylops ovinae*

**Figure 2:**
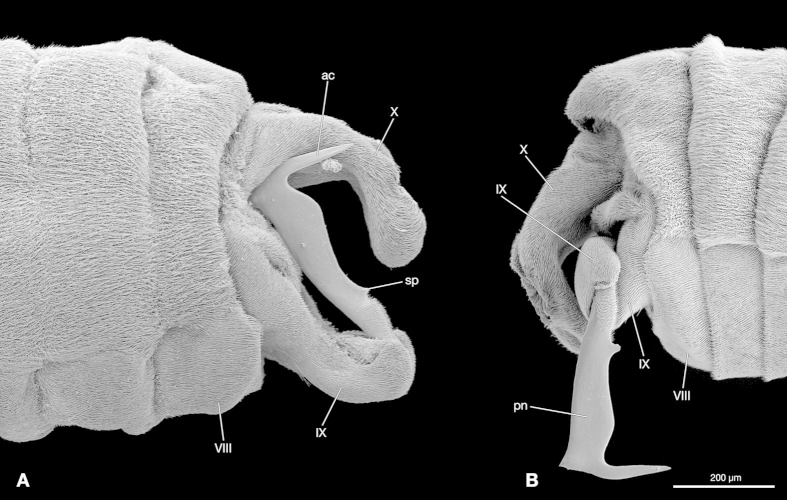
External male genital structures of *S. ovinae*, lateral view. (**A**) Penis in the resting state. (**B**) Penis unfolded. ac, acumen; IX, abdominal segment IX; pn, penis; sp, spike; VIII, abdominal segment VIII; X, abdominal segment X. SEM micrographs.

Abdominal segment IX (genital segment) is anteriorly retracted into abdominal segment VIII and extended posteriorly. It narrows towards the caudal tip of the abdomen. The 400 μm long penis arises on the caudo-dorsal end of the genital segment (Fig. 2). The penis is movably connected to the genital segment by a membrane and paired lateral joints. In the resting state it is folded beneath segment X (Fig. 2A). Its basal part is strongly sclerotized, broad and covered with microtrichia. The remaining ¾ are strongly compressed laterally and the surface is glabrous, without microtrichia and sensory hairs. The anterior margin is straight. The caudal border is curved and a small spike is present just above the base. The apical part is 130 μm long and bent at an angle of about 90° caudally. The phallotrema opens on its ventral side.

### Penetration site

**Figure 3:**
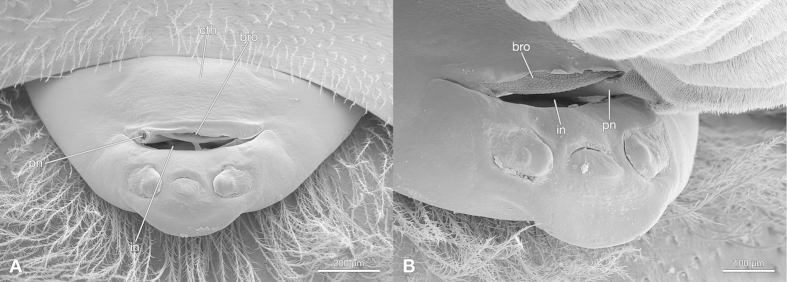
In copula fixed *S. ovinae*. (**A**) Female cephalothorax with penis broken off in the invagination. (**B**) Detail of the penetration. bro, birth opening; cth, cephalothorax of female; in, cephalothoracic invagination; pn, penis. SEM micrographs.

**Figure 4:**
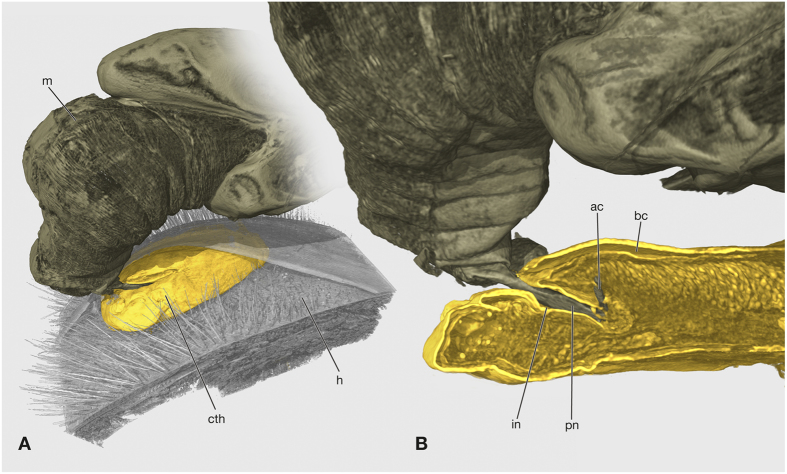
Volume render of the copulation of *S. ovinae*. (**A**) Overview, only the abdomen and part of the metathorax of the male is shown. (**B**) Detail, medio sagittal section of female cephalothorax with the penis inserted in the invagination and penetrating the cuticle of the female. ac, acumen; bc, brood canal; cth, cephalothorax of female; h, host; in, cephalothoracic invagination; m, male; pn, penis.

**Figure 5:**
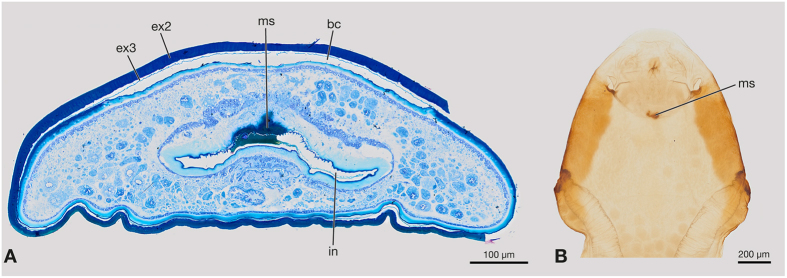
Mated females of *S. ovinae*. (**A**) Cross section of the cephalothorax at the level of the cephalothoracic invagination, ventral (physiological dorsal) is toward the top. (**B**) Cephalothorax with removed last larval exuvium, ventral view. bc, brood canal; ex2, exuvium of second instar larva; ex3, exuvium of third instar larva; in, cephalothoracic invagination; ms, mating scar.

During copulation the penis penetrates the thin cuticular closure of the invagination in front of the birth opening (Fig. 3). It is inserted laterally, either on the right or left side. The apical part of the penis perforates the ventral cuticle of the invagination in its posterior third (Fig. 4B). The sperm is injected into the hemocoel of the female. One day after mating, the penetration area is marked by a melanised spot on the cuticle in living females (Fig. 5B). In histological sections the spot appears dark blue and a dark blue amorphous structure is visible in the lumen of the invagination (Fig. 5A).

### Mating sequence

**Figure 6:**
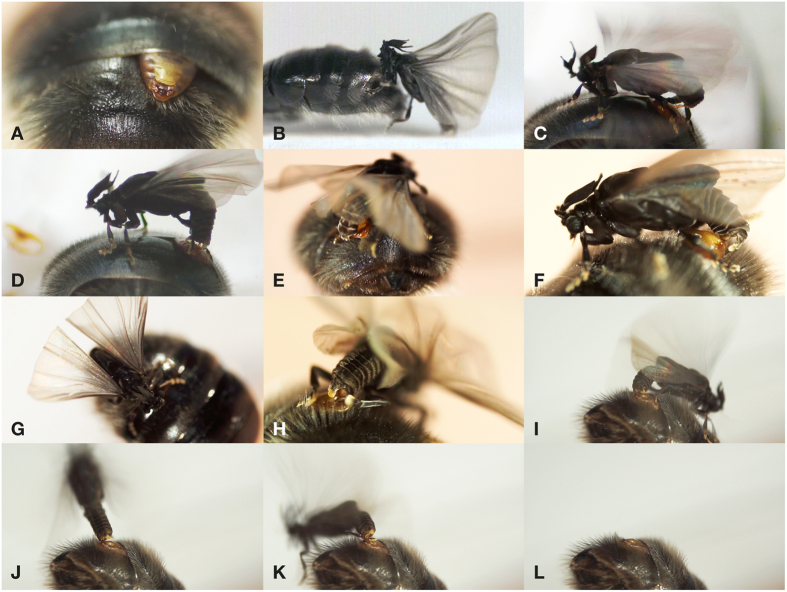
Mating of *S. ovinae* (film stills). (**A**) Abdomen of *A. vaga,* parasitized by a female. (**B**) Mounting the host. (**C**) Unfolding the penis. (**D**) Penetration. (**E–H**) Anchoring. (**I–L**) Separation.

Mating in *S. ovinae* shows a characteristic pattern, divided into five distinct stages (4 video recorded mating events analysed, the duration [in seconds] of the stages is given in parentheses in the following order: mean, minimum-maximum): 1. The male mounts the parasitized host bee (2, 2–2) (Fig. 6B,C). 2. It occupies a suitable position on the abdomen (4, 3–5). 3. The invagination is perforated (27, 7–63) (Fig. 6D). 4. The penis is anchored (683, 288–1058) (Fig. 6E–H). 5. The male separates from the female and leaves the host (9, 3–22) (Fig. 6I–L). The females are motionless before, during and after copulation in contrast to the behaviour described in females of *Xenos peckii*^26^. These females inflate their cephalothorax, and super-extrude it from the host wasp abdomen prior to mating, and retract it after the copulation. A detailed description of the mating sequence (Supplementary data) and two video clips (Video Clip S1, Video Clip S2) can be found in the supplementary material.

### Duration and frequency of copulation

**Figure 7:**
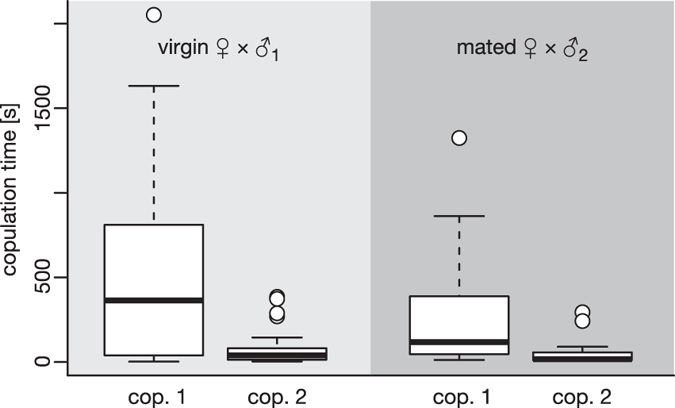
Duration of copulations of virgin and mated female *Stylops*. Boxes represent the interquartile range between first and third quartiles and the line inside represents the median. Whiskers denote the lowest and highest values within 1.5× interquartile range from the first and third quartiles, respectively. Circles represent outliers beyond the whiskers. cop. 1–2, copula 1–2.

We observed a total of 227 matings. The duration of all observed virgin matings (movie data not included) ranged between 2 s and 34 min 12 s (n = 114), their distribution was not normal, but right-tailed (Shapiro-Wilk test, P < 0.001 for all cases). Males can mate several times with the same female, in one case up to 14 times. In repeated matings of the same male with the same female the duration decreases considerably. A further significant decrease in duration occurs when a second male copulates with an already mated female (Mann-Whitney U test, P = 0.048 for the first copula) (Table 1, Fig. 7). The duration of the second copula is significantly shorter than the first one as shown by the Wilcoxon test (n = 31 paired observations for virgin, P = 0.022; n = 21 paired observations for mated, P = 0.001) as well as by the Mann-Whitney U test (n1 = 68, n2 = 31 unpaired observations for virgin, P < 0.001; n1 = 49, n2 = 21 unpaired observations for mated, P < 0.001). Raw data of the duration and frequency of copulation can be found in Supplementary Table 1.

### Duration of female attractiveness

Copulation with a second male took place between 50 min and 1 h 15 min after the first mating in five out of six females (n = 6). The female not performing a second copulation was repeatedly visited by a male. In between 1 h 31 min to 1 h 44 min four males copulated for a second time with the previous mated females (n = 6). In both cases where a second copula did not take place the females were completely ignored by the males. The attractiveness of mated females declines about 2 h after the first copula. This prevents further mating. Copulation did not take place from 2 h 9 min to 3 h 18 min (n = 5) (see Supplementary Table 2). However, two females were repeatedly visited by the males. These interactions involved contact with the maxillary palps and tarsi.

## Discussion

In Strepsiptera traumatic insemination was only confirmed for Mengenillidae^8^,^30^,^31^, while the mode of insemination in the Stylopidia was controversial. In contrast to previous studies, we are now able to demonstrate it also in a family of Stylopidia, the Stylopidae. In Mengenillidae, the penis can penetrate any part of the female’s body except for the head and the spermatozoa are injected directly in the hemocoel of the female^8^,^30^,^31^,^32^. A statement of Cook^33^ that the genital opening is used for fertilization is very likely a translation error citing from Silvestri^8^,^31^,^32^, who published in Italian. In contrast, *S. ovinae* males penetrate the cuticle of the invagination in front of the birth opening of the female’s cephalothorax.

Traumatic insemination in Strepsiptera may have evolved in a similar way as in Cimicomorpha (Heteroptera), *Drosophila* (Diptera), and *Harpactea* (Araneae), where it is likely linked with sperm competition, resulting in a shortcut of the female genital tract and sperm storage organs and thereby avoiding pre- and postcopulatory female choice mechanisms^5^. Tatarnic *et al.*^5^ suggested that traumatic insemination in Strepsiptera is likely a by-product of their parasitic lifestyle. We do not follow this interpretation as the females of the most basal lineage (Mengenillidae) are free-living. Instead, traumatic insemination in strepsipterans may have also evolved in the context of sperm competition in the first place. The disuse of the female genitalia could then drive the extreme reduction of these organs, with females lacking a vagina, genital chamber, bursa copulatrix, and receptaculum seminis, and the eggs floating freely in the hemolymph.

**Figure 8:**
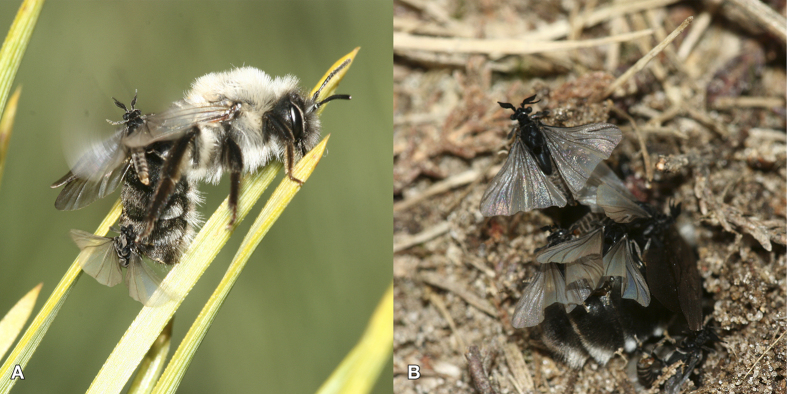
Mating of *S. ovinae*. **A**) Two males on a parasitized *A. vaga* (Osnabrück, 10.ii.2008). (**B**) Five males on a parasitized host (Osnabrück, 23.ii.2008). Photographs^©^ W. Rutkies.

Prolonged copulation, where males retain genital contact considerably longer after the insemination of the female, is reported for many insect groups^34^. Alcock^34^ explained this behaviour as a way in which a male might reduce the chance that the female receives sperm from other males. Copulation in strepsipterans is usually short and ranges between 1 s (*Elenchus*) and 5 min (*Corioxenos*) in most cases (Table 2). *Stylops ovinae* is an exception with a prolonged copulation, with an average duration of 8 min and a maximum of 34 min. A brief mating of a few seconds is sufficient for males of this species to transfer enough sperm to fertilize all of the eggs. Insemination occurs at the beginning of the copulation^25^,^35^, and shortly after the outset (2.5 to 3 min) the spermatozoa are already distributed throughout the entire abdomen of the female^25^. Lauterbach^25^ ascribed a prolonged copulation in *S. ovinae* to an involuntary entanglement of the penis in the cephalothorax. We observed this only as very exceptional cases. An explanation for the prolonged copulation in *S. ovinae* could be that, unlike in other strepsipterans, hatching of males of *S. ovinae* is limited to a few days per year (s. b). Therefore often several males compete for one female (Fig. 8). Prolonged mating possibly reduces competition between their own sperm and sperm from other males. However, fertilization processes could not be detected on semi-thin sections of *S. ovinae* females fixed 1 h after the copula^25^. Nevertheless, sperm cells are difficult to find in oocytes with light microscopy^10^. Transmission electron microscopic examinations of females of *X. vesparum* (fixed 1–2 h after mating) showed oocytes surrounded by numerous spermatozoa and a single sperm cell was found within the cytoplasma of an oocyte^10^. No females were fixed directly after the copula.

When females mate more than once, the relative number of sperm cells can determine the number of their offspring^36^,^37^. Therefore males of many insects adjust copulation duration and also adapt the volume of the ejaculate in relation to the female mating status because of the need to conserve sperm (e.g. Siva-Jothy & Stutt^38^). Our results show that male *S. ovinae* detect the mating status of the females, as the second mating with the same female is significantly shorter, and the duration decreases further significantly when a second male mates with an already mated female. It is likely that the males detect the mating status with apical sensilla on the maxillary palps and/or sensory spots on the tarsi as they usually touch the cephalothorax with the tips of the maxillae first and then with the tarsi (s. Mating sequence). A similar behaviour was also observed in *Xenos peckii*, but in this case not involving the palps^26^. It can be excluded that the mating status is detected with the copulatory organ as it is described for bed bugs^38^ because the tip of the penis of *Stylops* completely lacks sensilla. Substances, which could play a role in this context, are the ejaculate of the male, the emergence of hemolymph from the penetration site, pheromones released by the males on the cephalothorax and the host’s abdomen during copulation, or pheromones released by the females indicating that they have mated. Whether males of *Stylops* adapt the quantity of sperm to the mating status of the female is unknown.

Mechanical damage resulting from traumatic insemination can cause costs for females^6^,^39^,^40^,^41^. Females of the bed bug *Cimex lectularius* (Heteroptera, Cimicidae) have evolved a novel paragenital organ, the so-called spermalege, where the males penetrate the cuticle. The cuticle of this specialized region on abdominal sternite V is thickened and rich in resilin^41^. This counter-adaptation efficiently reduces the costs of the unusual mode of sperm transfer^6^,^41^. In contrast to all other strepsipteran males, those of *S. ovinae* often hatch in masses and usually only during few days in late winter/early spring^25^,^27^,^35^. Sometimes males occur only on a single day of the year^28^. Since the females of *Stylops* are attractive for males about 2 hours after the first copula (s. a.), multiple mating could lead to increased trauma to females. This sexual conflict probably led to the evolution of the cephalothoracic invagination where the cuticle of the females is penetrated. It can be hypothesised that this novel paragenital organ reduces the costs of the traumatic insemination in *Stylops*. This is similar to the spermalege in bed bugs and thus provides an interesting example of convergent evolution within the context of traumatic insemination and sexual selection.

The confirmed traumatic insemination in a strepsipteran representative with endoparasitic females does not refute the hypothesis that the switch to an obligatory endoparasitic life style of the females (Stylopidia) resulted in a change to a non-traumatic insemination (brood canal mating). The occurrence of spermatozoa along both the brood canal and birth organs suggests – at least in *X. vesparum* – brood canal mating, although the authors do not exclude that some sperm cells might escape into the brood canal during traumatic insemination, leaving open the insemination question^10^. However, as the basal lineage with free-living females also uses traumatic insemination, we prefer the hypothesis of Silvestri^42^,^43^ that the original mode of traumatic insemination was maintained during the transition from free-living (Mengenillidae) to endoparasitic females (Stylopidia). Contrary to the view of Kathirithamby *et al.*^7^ a change in Stylopidia to a non-traumatic insemination (groundplan) and again to a traumatic mode in *Stylops* appears highly unlikely.

## Methods

### Study organisms

A total of 150 mining bees *Andrena vaga* (Hymenoptera, Apidae) parasitized by *S. ovinae* were collected near Osnabrück in the sand pit Niedringhaussee (Germany), on 27.02.2013, 10.03.2013, 11.01.2014, and 10.02.2014. Parasitized *A. vaga* appear before non-parasitized individuals at a soil temperature of 8 °C during daytime, and males of *S. ovinae* hatch shortly after that^25^. Therefore the host bees were dug out before their first appearance. Until fixation or observation and recording of the mating, the bees were kept dark at 4 °C in glass vessels (0.5 l) closed with gauze and half filled with moist sand.

### Recording of mating events

Four mating events of *S. ovinae* were recorded with 25–30 frames/s with a Cam One Infinity HD (Dakine, Rietburg, Germany) and a Sony α NEX-5N (Sony Corporation, Tokyo, Japan) connected to a bellows with extension tubes and Carl Zeiss magnifier lens (Luminar 40, 63, 100 mm, Carl Zeiss, Oberkochen, Germany). For lighting a cold light source (KL 750, Schott, Mainz, Germany) was used. Single images were taken from the movies using the software VLC player. The copulations were initiated in transparent plastic trays (4 cm diameter, 1 cm high) at 21 ± 1 °C. Covering the trays with a glass plate prevented the escape of the males. To avoid damage to the males by bites of the hosts and dispensing of the meconium of the host bees, the abdomen of the hosts was removed. The abdomen was either attached directly to modelling clay with its anterior end on the bottom of the trays, or its ventral side was glued to an insect pin (UHU Alleskleber Super, Germany) and fixed in the clay. Two individuals of *A. vaga* were parasitized each with a virgin female and a male puparium. Hatching of the males was induced by the light stimulus and increase in temperature. Two individuals of *A. vaga* were only parasitized by one virgin female *S. ovinae* and a freshly hatched male was placed in each plastic tray.

Two matings of *S. ovinae* were recorded with a FASTCAM-1024PCI high-speed video camera (Photron, San Diego, USA) with 1000 frames per second equipped with an AF Micro Nikkor 60 mm lens (Nikon, Düsseldorf, Germany). For lighting and temperature see above. The abdomen of two *A. vaga*, each parasitized with a virgin female *Stylops*, were fixed to an insect pin with superglue inside a glass container (20.5 × 12.5 × 14.5 cm). The head and terminal section were covered with gauze. At the top end of the container a computer fan generated a weak airflow to produce a pheromone gradient in the vessel. For each recording, one male was placed in the end section of the container.

### Mating experiments

In order to determine the duration and frequency of copulation, 68 intact specimens of *A. vaga*, each parasitized with a single virgin female, were placed separately in glass vessels (0.5 l) with absorbent paper to prevent sticking of the males by excretions of the host bees at 21 ± 1 °C. Lighting was carried out with a cold light source (s. a.). A freshly hatched male was placed in each glass vessel. After the first copulation the males were left in the vessel for ca. 10 minutes. Thereafter, the first male was removed and a second freshly hatched male was placed to 58 individuals of the females.

To assess the duration of female attractiveness after copulation, we confronted 17 *A. vaga* individuals with one newly hatched male, each of the hosts parasitized with a single female from 50 min to 3 h 18 min after the first copulation (glass vessels, temperature and lighting as described above).

### In copula fixation

Copulations were initiated, as described above (s. recording of mating events), and a male was placed in the tray. Thirty seconds after the initiation of mating the couples were fixed with ca. 2 ml of 100% ethanol cooled to −80 °C and then frozen at −80 °C for 2 weeks. After thawing, the specimens were transferred into fresh 100% ethanol (1 h, three times alternatingly). Twelve pairs of *S. ovinae* fixed in copula were obtained. Three copulae were critical point dried, as well as a female with a broken penis in the cephalothoracic invagination, the corresponding male with missing penis, and a male with unfolded penis (Leica EM CPD300, Leica, Wetzlar, Germany).

### Scanning electron microscopy

One copula of *S. ovinae*, the female with the penis in the cephalothoracic invagination, the male with unfolded penis, and one male with the penis in the resting position were glued on a fine pin with nail polish and mounted on a rotatable specimen holder^44^. The specimens were sputter coated with gold (Sputter Coater, sample preparation division, Quorum Technologies Ltd., Ashford, England) and examined in an ESEM XL30 (Philips, Amsterdam, The Netherlands). Scandium FIVE software (Olympus, Münster, Germany) was used for obtaining high-resolution images. In order to obtain a higher depth of field, several images of selected views were taken at different focal planes and assembled with Helicon Focus Version 4.2.7 (Helicon Soft, Kharkov, Ukraine).

### Histology

For serial sections, the females were extracted from the host, fixed in Dubosq Brasil, dehydrated in an ascending ethanol series and embedded in Araldite CY-212 (Agar Scientific, Stansted/Essex, England). This included two mated specimens (cross-sections) and one virgin (longitudinal sections). The serial sections (1 μm) were carried out with a HM 360 (Microm, Walldorf, Germany) microtome with a diamond knife. They were stained with Toluidine blue and Pyronin G (Waldeck GmbH & Co. KG/Division Chroma, Münster, Germany). Individual sections were documented with an Olympus dot.Slide microscope (BX51, software version 3.4, Olympus, Tokyo, Japan).

### Micro-computed tomography (μCT) and 3D reconstruction

Micro-CT scans of fixed copulae were performed with a Skyscan 1172 desktop micro-CT scanner (Bruker Micro-CT, Kontich, Belgium). This resulted in a volumetric dataset with isometric voxels that had an edge-length of 1.67 microns. Based on one of the μCT-image stacks the cephalothorax of the female and the abdomen of the male were reconstructed three-dimensionally using Visage Imaging Amira 5.3 software (Visage Imaging GmbH, Berlin, Germany) and VGStudio MAX (Volume Graphics, Heidelberg, Germany).

### Statistical analyses

The distribution of copulation durations (separately for each group and each copula) was tested for normality using a Shapiro Wilk test. Differences in copulation times between groups were tested using the Mann-Whitney U test. Differences between duration of first and second copulation were tested by Wilcoxon test for those individuals were both durations were observed as well as by Mann-Whitney U test for all individuals as independent observations. All calculations were performed using the software SPSS v21^45^.

### Images

All image plates were prepared using Adobe Photoshop and Illustrator CS4 (Adobe, San Jose, USA).

### Terminology

For the female anatomy and especially for structures related to mating and birth we use the terminology established by Lauterbach^25^ and Pohl^46^, and the terminology of Hünefeld *et al.*^47^ for postabdominal structures of males.

## Additional Information

**How to cite this article**: Peinert, M. *et al.* Traumatic insemination and female counter-adaptation in Strepsiptera (Insecta). *Sci. Rep.* **6**, 25052; doi: 10.1038/srep25052 (2016).

## Supplementary Material

Supplementary Information

Supplementary Video S1

Supplementary Video S2

## Figures and Tables

**Table 1:** Duration and frequency of copulation in *S. ovinae*.

		Virgin females (n = 68)			Mated females (n = 49)		
		first copula (n = 68)	second copula (n = 31)	third copula (n = 15)	first copula (n = 49)	second copula (n = 21)	third copula (n = 6)
Duration of copula (min:s)	average	08:03	01:19	00:35	04:23	00:53	00:16
	min.	00:02	00:03	00:05	00:12	00:05	00:06
	max.	34:12	06:25	06:25	22:04	04:54	00:38
	median	03:29	00:39	00:15	01:57	00:16	00:12

**Table 2:** Duration of copulations in strepsipteran species.

Species	Duration of copulations	Reference
*Corioxenos antestiae*	a few seconds to 1 min, seldom 5 min	23
*Elenchus tenuicornis*	1–3 s	24
*Halictophagus silwoodensis*	a few seconds	48
*Xenos peckii*	20–50 s	9
*Xenos peckii*	5 s	26
*Xenos vesparum*	5–15 s	10
*Stylops ovinae*	a few seconds to 2 min, rarely 5 min. In exceptional cases, 20 min and more	35
*Stylops ovinae*	2 s to 34 min 12 s	present study
*Stylops pacifica*	2 min 15 s	49

## References

[b1] Reinhardt K., Anthes N. & Lange R. Copulatory wounding and traumatic insemination. Cold Spring Harb. Perspect. Biol. 7, a017582 (2015). 25877218 10.1101/cshperspect.a017582 PMC4448625

[b2] Řezáč M. The spider *Harpactea sadistica*: co-evolution of traumatic insemination and complex female genital morphology in spiders. Proc. R. Soc. B. 276, 2697–2701 (2009). 10.1098/rspb.2009.0104 PMC2839943 19403531

[b3] Kamimura Y. Twin intromittent organs of *Drosophila* for traumatic insemination. Biol. Lett. 3, 401–404 (2007). 17519186 10.1098/rsbl.2007.0192 PMC2391172

[b4] Mattei A. L., Riccio M. L., Avila F. W. & Wolfner M. F. Integrated 3D view of postmating responses by the *Drosophila melanogaster* female reproductive tract, obtained by micro-computed tomography scanning. P. Natl. Acad. Sci. USA 112, 8475–8480 (2015). 10.1073/pnas.1505797112 PMC4500220 26041806

[b5] Tatarnic N. J., Cassis G. & Siva-Jothy M. T. Traumatic insemination in terrestrial arthropods. Annu. Rev. Entomol. 59, 245–261 (2014). 24160423 10.1146/annurev-ento-011613-162111

[b6] Morrow E. H. & Arnqvist G. Costly traumatic insemination and a female counter-adaptation in bed bugs. Proc. R. Soc. B. 270, 2377–2381 (2003). 10.1098/rspb.2003.2514 PMC1691516 14667354

[b7] Kathirithamby J. *et al.* We do not select, nor are we choosy: reproductive biology of Strepsiptera (Insecta). Biol. J. Linn. Soc. 116, 221–238 (2015).

[b8] Silvestri F. Studi sugli ‘Strepsiptera’ (lnsecta). III. Descrizione e biologia di 6 specie italiane di *Mengenilla. Boll. Lab. Zool*. gen. Fac. Agrar. Portici 32, 197–282 (1943).

[b9] Schrader S. H. Reproduction in *Acroschismus wheeleri* Pierce. J. Morphol. 39, 157–205 (1924).

[b10] Beani L. *et al.* Mating of *Xenos vesparum* (Rossi) (Strepsiptera, Insecta) revisited. J. Morphol. 265, 291–303 (2005). 16047336 10.1002/jmor.10359

[b11] Kristensen N. P. Phylogeny of insect orders. Annu. Rev. Entomol. 26, 135–157 (1981).

[b12] Pohl H. & Beutel R. G. The Strepsiptera-Odyssey: the history of the systematic placement of an enigmatic parasitic insect order. Entomologia 1, e4 (2013).

[b13] Boussau B. *et al.* Strepsiptera, phylogenomics and the long branch attraction problem. Plos ONE 9, e107709 (2014). 25272037 10.1371/journal.pone.0107709 PMC4182670

[b14] Beutel R. G. *et al.* Morphological and molecular evidence converge upon a robust phylogeny of the megadiverse Holometabola. Cladistics 27, 341–355 (2011). 10.1111/j.1096-0031.2010.00338.x 34875787

[b15] Niehuis O. *et al.* Genomic and morphological evidence converge to resolve the enigma of Strepsiptera. Curr. Biol. 22, 1309–1313 (2012). 22704986 10.1016/j.cub.2012.05.018

[b16] Misof B. *et al.* Phylogenomics resolves the timing and pattern of insect evolution. Science 346, 763–767 (2014). 25378627 10.1126/science.1257570

[b17] Brues C. T. A contribution to our knowledge of the Stylopidae. Zool. Jb. (Anat., Ontog.) 18, 241–270 (1903).

[b18] Nassonov N. V. Untersuchungen zur Naturgeschichte der Strepsipteren. – Aus dem Russischen übersetzt von Alexander v. Sipiagin. Mit Anmerkungen und einem kritischen Anhang über einige Ansichten Meinerts betreffs der Anatomie des Weibchens. S. J.-Ber. naturw.-med. Ver. Innsbruck 33, I–Vlll, 1–206 (1910).

[b19] Siebold C. T. E. Über Strepsiptera. Arch. Naturgesch. 9, 137–162 (1843).

[b20] Sagemehl M. Ein Paar von *Stylops* sp. in der Begattung. Sber. naturf. Ges. Dorpat 6, 399–400 (1884).

[b21] Muir F. Notes on some Fijian insects. Rep. exp. Hawaii. sugar planters’ Assoc. Expt. Sta. 2, 3–11 (1906).

[b22] Hofeneder K. *Stylops* in copula. Verh. zool.-bot. Ges. Wien 73, 128–134 (1923).

[b23] Kirkpatrick T. Studies on the ecology of coffee plantations in East Africa. II. The autecology of *Antestia* spp. (Pentatomidae) with a particular account of a Strepsipterous parasite. [Part. II: The bionomics of *Corioxenos antestiae*. Blair]. Trans. R. Entomol. Soc. Lond. 86, 247–343 [281–341] (1937).

[b24] Lindberg H. Der Parasitismus der auf *Chloriona*-Arten (Homoptera Cicadina) lebenden Strepsiptere *Elenchinus chlorionae* n. sp. sowie die Einwirkungen derselben auf ihren Wirt. Acta Zool. Fenn. 22, 1–179 (1939).

[b25] Lauterbach G. Begattung und Larvengeburt bei den Strepsipteren. Zugleich ein Beitrag zur Anatomie der *Stylops*-Weibchen. Z. Parasitenkd. 16, 255–297 (1954). 13227330 10.1007/BF00260193

[b26] Hrabar M., Danci A., McCann S., Schaefer P. W. & Gries G. New findings on life history traits of *Xenos peckii* (Strepsiptera: Xenidae). Can. Entomol. 146, 514–527 (2014).

[b27] Tolasch T., Kehl S. & Dötterl S. First sex pheromone of the order Strepsiptera: (3R,5R,9R)-3,5,9-Trimethyldodecanal in *Stylops melittae* Kirby, 1802. J. Chem. Ecol. 38, 1493–1503 (2012). 23224569 10.1007/s10886-012-0215-6

[b28] Lagoutte R. *et al.* Total synthesis, proof of absolute configuration, and biosynthetic origin of stylopsal, the first isolated sex pheromone of Strepsiptera. Chem. Eur. J. 19, 8515–8524 (2013). 23630024 10.1002/chem.201204196

[b29] Dallai R. *et al.* Fine structure of the Nassonow’s gland in the neotenic endoparasitic of female *Xenos vesparum* (Rossi) (Strepsiptera, Insecta). Tissue Cell 36, 211–220 (2004). 15140598 10.1016/j.tice.2004.02.001

[b30] Parker H. L. & Smith H. D. Further notes on *Eoxenos laboulbenei* Peyerimhoff with a description of the male. Annals Ent. Soc. Am. 27, 468–479 (1934).

[b31] Silvestri F. Studi sugli ‘Strepsiptera’ (lnsecta). I. Ridescrizione e ciclo dell’*Eoxenos laboulbenei* Peyerimoff. *Boll. Lab. Zool*. gen. Fac. Agrar. Portici 31, 311–341 (1941).

[b32] Silvestri F. Descrizione della femina e del maschio di una nuova specie di *Mengenilla* Hofeneder (Strepsiptera). *Boll. Lab. Zool*. gen. Fac. Agrar. Portici 28, 1–10 (1933).

[b33] Cook J. L. Review of the biology of parasitic insects in the order Strepsiptera. Comp. Parasitol. 81, 134–151 (2014).

[b34] Alcock J. Postinsemination associations between males and females in insects: the mate-guarding hypothesis. Annu. Rev. Entomol. 39, 1–21 (1994).

[b35] Grabert B. Bau der Geschlechtsorgane und Kopulation beim Stylops-Männchen (Insecta, Strepsiptera). 1–40 (FU Berlin, 1953).

[b36] Parker G. A. Sperm competition games – sneaks and extra-pair copulations. Proc. R. Soc. B. 242, 127–133 (1990).

[b37] Ball M. A. & Parker G. A. Sperm competition games: a comparison of loaded raffle models and their biological implications. J. theor. Biol. 206, 487–506 (2000). 11013110 10.1006/jtbi.2000.2142

[b38] Siva-Jothy M. T. & Stutt A. D. A matter of taste: direct detection of female mating status in the bedbug. Proc. R. Soc. B. 270, 649–652 (2003). 10.1098/rspb.2002.2260 PMC1691276 12769466

[b39] Arnqvist G. & Nilsson T. The evolution of polyandry: multiple mating and female fitness in insects. Anim. Behav. 60, 145–164 (2000). 10973716 10.1006/anbe.2000.1446

[b40] Tatarnic N. J. & Cassis G. Sexual coevolution in the traumatically inseminating plant bug genus *Coridromius*. J. Evolution. Biol. 23, 1321–1326 (2010). 10.1111/j.1420-9101.2010.01991.x 20456571

[b41] Michels J., Gorb S. N. & Reinhardt K. Reduction of female copulatory damage by resilin represents evidence for tolerance in sexual conflict. J. Roy. Soc. Interface 12, 20141107 (2015). 25673297 10.1098/rsif.2014.1107 PMC4345479

[b42] Silvestri F. Sulla maniera di fecondazione della femmina negli ‘Stylopidae’ (lnsecta, Strepsiptera). Atti. R. Accad. Ital. Rc. C1. sci. fis. mat. nat. 7, 553–556 (1940).

[b43] Silvestri F. Studi sugli ‘Strepsiptera’ (lnsecta). – II. – Descrizione, biologia e sviluppo postembrionale dell’*Halictophagus tettigometrae* Silv. *Boll. Lab. Zool*. gen. Fac. Agrar. Portici 32, 11–48 (1941).

[b44] Pohl H. A scanning electron microscopy specimen holder for viewing different angles of a single specimen. Microsc. Res. Tech. 73, 1073–1076 (2010). 20196104 10.1002/jemt.20835

[b45] IBM Corporation (2012). IBM SPSS Statistics for Windows, Version 21.0. Armonk, NY, USA. URL http://www.ibm.com/

[b46] Pohl H. & Beutel R. G. The phylogeny of Strepsiptera (Hexapoda). Cladistics 21, 328–374 (2005). 10.1111/j.1096-0031.2005.00074.x 34892965

[b47] Hünefeld F., Pohl H., Wipfler B., Beckmann F. & Beutel R. G. The male postabdomen and genital apparatus of †*Mengea tertiaria*, a strepsipteran amber fossil (Insecta). J. Zool. Syst. Evol. Res. 49, 298–308 (2011).

[b48] Henderickx H. Faunistische bemerkingen over Strepsiptera met onderzoek van een populatie *Halictophagus silwoodensis* (Halictophagidae) in het Nationaal Park Hoge Kempnen (Maasmechelen). Phegea 36, 103–107 (2008).

[b49] Linsley E. & MacSwain J. Observations on the habits of *Stylops pacifica* Bohart. Univ. Calif. Pub. Entomol. 11, 395–430 (1957).

