# Supplementary material for: Traumatic insemination and female counter-adaptation in Strepsiptera (Insecta)
Source: Sci Rep. 2016 Apr 29;6:25052. doi: 10.1038/srep25052 (PMC4850473; doi:10.1038/srep25052)
Supplement: Supplementary Information [file srep25052-s1.pdf]

Supplementary Information

Traumatic insemination and female counter-adaptation in Strepsiptera (Insecta)

Miriam Peinert[1], Benjamin Wipfler[1], Gottfried Jetschke[2], Thomas Kleinteich[3], Stanislav N. Gorb[3], Rolf G. Beutel[1], and Hans Pohl[1]*

[1]Entomology Group, Institut für Spezielle Zoologie und Evolutionsbiologie mit Phyletischem Museum, Friedrich-Schiller-Universität Jena, Erbertstrasse 1, D-07743 Jena, Germany

[2]Institut für Ökologie, Friedrich-Schiller-Universität Jena, Dornburger Strasse 159, D-07743 Jena, Germany

[3]Department of Functional Morphology and Biomechanics, Institute of Zoology, Christian-Albrechts-Universität zu Kiel, Am Botanischen Garten 1–9, D-24118 Kiel, Germany

Video legends

Video Clip S1. Compilation of the mating sequence of *Stylops ovinae*. Video replay is at 100 % real-time speed.

Video Clip S2. Walking approach, mounting the host and penetration of a *Stylops ovinae* male. Video is recorded with 1000 fps and replay is at 30 fps (3 % real-time speed).

Supplementary data. Detailed description of the mating sequence.
The duration (in seconds) of the stages is given in parentheses in the following order: mean, minimum – maximum:

1. Mounting the host (2, 2–2) (Fig. 6B, C). The male identifies the female a few seconds after hatching or being placed in the tray. It approaches the parasitized bee walking on the ground. In the typical case it climbs onto the abdomen from behind and folds back the antennae and maxillae.

2. Positioning on the abdomen (4, 3–5). The first contact with the cephalothorax of the female is usually achieved by the tips of the male's maxillae. Thereafter, the tarsi of the fore or middle legs touch the female and the frequency of the wing beat is lowered at the same time. As soon as the hind tarsi are in contact, their proximal tarsomeres clasp the side of the cephalothorax. Meanwhile, the male turns to the right (about 45° to the longitudinal axis of the host), if the cephalothorax is extruded on the left side of the host abdomen, or to the left, when the female is extruded on the right side.

3. Penetration (27, 7–63) (Fig. 6D). The penis is unfolded shortly before the penetration. The ventral part of segment IX is twisted at the base and the segment bends to the left or right at an angle of approximately 90°. Thereby either the right or left side of the segment is facing towards the rear. By twisting segment IX, the penis is unfolded downwards at an angle of approximately 180° (Fig. 2B). The penetration is followed by several protractions of the abdomen (2–13). It is flexed ventrad towards the thorax at an angle of ca. 90°. The protractions are accompanied by torsions of the abdomen, and the male is moving slightly forward.

4. Anchoring (683, 288–1058) (Fig. 6E-H). The halteres and hind wings do not move at this stage, except for slight trembling. The antennae and maxillae are still folded backwards and the legs move independently with different frequencies. The fore tarsi are placed on the smooth marginal regions of abdominal tergites III and IV of the host bee. They usually move infrequently (1–2 times per min). The mid legs are usually placed on hairy regions of the tergites III and IV. With 8–161 twitches per minute they move with a distinctly higher frequency than the fore legs. The hind legs are always placed right and left of the female cephalothorax, but do not clasp it. They move with the highest frequency (146–203 beats per min). The adhesive pads of the males do not attach to the hairy tergites of the host bee. The tarsi rather bend the hairs.

5. Separation (9, 3–22) (Fig. 6 I-L). After mating, the male separates from the female and detaches its tarsi from the host surface. It actively beats with the halteres and hind wings and alternatively turns to the left and right. By the wing beat created propulsion and the torsion of the abdomen, the penis is pulled out mechanically from the invagination of the female.

Supplementary table 1. Raw data of the duration and frequency of copulation. No second male was placed to females 2, 4, 6, 8, 10, 11, 13, 14, 27, and 70.

	Female ID	Virgin females Nr. of copula (duration [s])				Mated females Nr. of copula (duration [s])			
**Date**		**1**	**2**	**3**	**total nr. of cop.**	**1**	**2**	**3**	**total nr. of cop.**
25.01.2014	2	643	270	5	3				
25.01.2014	3	380			1	810			1
25.01.2014	4	600			1				
25.01.2014	5	1200			1	540			1
26.01.2014	6	917			1				
26.01.2014	7	958			1	404			1
26.01.2014	8	1185	56	65	8				
26.01.2014	9	956	33		2	1324			1
26.01.2014	10	1536			1				
26.01.2014	11	706			1				
28.01.2014	12	168	385		2	102	45		2
28.01.2014	13	65	78	30	14				
28.01.2014	14	1286	79	15	4				
28.01.2014	15	1301			1	251			1
28.01.2014	16	766			1	308			1
30.01.2014	17	24	83	61	3	46	13	9	3
30.01.2014	18	520			1	116	8		2
30.01.2014	19	153			1	30	11	6	3
30.01.2014	20	143	3	10	3	32	8		2
31.01.2014	21	12	373		2	117	57		2
31.01.2014	22	77			2	94	15	6	5
01.02.2014	23	1632			1	42			1
01.02.2014	24	585	109		2	453			1
01.02.2014	25	34			1	813	50		2
01.02.2014	26	683	43		3	29	294		2
03.02.2014	27	412			1				
20.02.2014	29	27			1	15			1
20.02.2014	30	133			1	388			2
20.02.2014	31	694	30		2	50	11	38	3
21.02.2014	32	651			1	312	71		2
21.02.2014	33	962			1	369	11		2
21.02.2014	34	209			1	62			1
21.02.2014	35	20	7		2	38	22		2
21.02.2014	36	900			1	80			1
23.02.2014	37	666			1	246	90	15	3
23.02.2014	38	30	10	15	3	287	82		2
23.02.2014	39	33	39	11	3	75	15	23	3
23.02.2014	40	20	288		2	287			1
23.02.2014	41	284	18		2	571			1
26.02.2014	42	21	51		2	58	14		2
26.02.2014	43	993			1	862			1
26.02.2014	44	672			1	462			1
03.03.2014	45	134			1	281			1
03.03.2014	46	813			1	724			1
04.03.2014	47	8	145		2	176	39		2
04.03.2014	48	99			1	13	5		2
04.03.2014	49	94	58		2	388	242		2
04.03.2014	50	347	44	187	3	54			1

Supplementary table 1. Continuation.

Date	Female ID	Virgin females Nr. of copula (duration [s])				Mated females Nr. of copula (duration [s])			
		**1**	**2**	**3**	**total nr. of cop.**	**1**	**2**	**3**	**total nr. of cop.**
04.03.2014	51	201	23	71	3	117			1
04.03.2014	52	769			1	13			1
11.03.2014	53	962			1	15			1
11.03.2014	54	40			1				
11.03.2014	55	2052			1	21	16		2
13.03.2014	56	33			1	58			1
13.03.2014	57	985			1	495			1
13.03.2014	58	808			1	786			1
28.03.2014	59	13	12		2	15			1
28.03.2014	60	395			1	12			1
28.03.2014	61	956			1	50			1
31.03.2014	62	135	15		2				
31.03.2014	63	8	10	7	5				
31.03.2014	64	39	5	10	4				
01.04.2014	65	551	20	22	3				
01.04.2014	66	61	16	12	3				
01.04.2014	67	72			1				
01.04.2014	68	10	124		2				
01.04.2014	69	15	9	7	3				
01.04.2014	70	2	5		2				

Supplementary table 2. Time after which a previously mated female was confronted with a newly hatched male and second copulation success (n = 17). x successful second copulation, – no second copulation.

Time after first mating (h:min)	0:50	1:01	1:00	1:00	1:00	1:15	1:31	1:33	1:33	1:36	1:39	1:44	2:09	2:20	2:25	3:12	3:18
Second copulation	x	x	x	x	–	x	–	x	x	x	x	–	–	–	–	–	–
